# Impact of COVID-19 on Global Kidney Transplantation Service Delivery: Interim Report

**DOI:** 10.3389/ti.2022.10302

**Published:** 2022-03-28

**Authors:** Vassilios Papalois, Camille N. Kotton, Klemens Budde, Julian Torre-Cisneros, Davide Croce, Fabian Halleck, Stéphane Roze, Paolo Grossi

**Affiliations:** ^1^ Renal and Transplant Directorate, Imperial College Healthcare NHS Trust, London, United Kingdom; ^2^ Infectious Diseases Division, Massachusetts General Hospital and Harvard Medical School, Boston, MA, United States; ^3^ Department of Nephrology and Medical Intensive Care, Charité Universitätsmedizin Berlin, Berlin, Germany; ^4^ Maimonides Institute for Biomedical Research of Cordoba (IMIBIC), Reina Sofía University Hospital, University of Cordoba, Cordoba, Spain; ^5^ CIBER of Infectious Diseases (CIBERINFEC), Instituto de Salud Carlos III, Madrid, Spain; ^6^ Research Centre in Health Economics and Healthcare Management, Carlo Cattaneo University, Castellanza, Italy; ^7^ Vyoo Agency, Health-Economics Department, Villeurbanne, France; ^8^ Department of Infectious Diseases, University of Insubria, Varese, Italy; ^9^ Infectious and Tropical Diseases Unit, ASST-Sette Laghi, Varese, Italy

**Keywords:** kidney transplantation, COVID-19 pandemic, cytomegalovirus management, service delivery, historical record, 2020

## Abstract

This article gives a personal, historical, account of the impact of the COVID-19 pandemic on transplantation services. The content is based on discussions held at two webinars in November 2020, at which kidney transplantation experts from prestigious institutions in Europe and the United States reflected on how the pandemic affected working practices. The group discussed adaptations to clinical care (i.e., ceasing, maintaining and re-starting kidney transplantations, and cytomegalovirus infection management) across the early course of the pandemic. Discussants were re-contacted in October 2021 and asked to comment on how transplantation services had evolved, given the widespread access to COVID-19 testing and the roll-out of vaccination and booster programs. By October 2021, near-normal life and service delivery was resuming, despite substantial ongoing cases of COVID-19 infection. However, transplant recipients remained at heightened risk of COVID-19 infection despite vaccination, given their limited response to mRNA vaccines and booster dosing: further risk-reduction strategies required exploration. This article provides a contemporaneous account of these different phases of the pandemic from the transplant clinician’s perspective, and provides constructive suggestions for clinical practice and research.

## Introduction

All aspects of health care are affected by the COVID-19 pandemic (1–3). In November 2020, kidney transplantation experts from Europe and the US attended two webinars to describe the challenges of COVID-19 and measures taken to maintain kidney transplantations during the pandemic. Discussants were re-contacted in late 2021, to reflect on the continuing situation. By then, vaccination, booster and antibody testing programs were widespread, despite substantial ongoing cases of COVID-19 infection.

## Kidney Transplantation Rates

In November 2020, kidney transplantation rates were generally near normal [Table 1 (4, 5)] after many institutions had reduced–or suspended–procedures (1, 2). Discussants estimated that, globally, kidney transplantation rates would be ∼20% lower in 2020 than expected, but individuals held varying views.

**TABLE 1:** Kidney transplantation rates in the US and Europe, 2014–2021 (4, 5).

	2021	2020	2019	2018	2017	2016	2015	2014
Deceased Donor, United States	13,214	11,925	11,152	9,867	9,401	9,116	8,250	7,763
Living Donor, United States	5,970	5,235	6,866	6,443	5,811	5,629	5,628	5,538
Deceased Donor, Eurotransplant^a^	2,933	2,831	3,161	3,480	3,093	3,278	3,424	3,348
Living Donor, Eurotransplant^a^	1,069	942	1,183	1,328	1,294	1,338	1,323	1,348

a Eurotransplant countries: Austria, Belgium, Croatia, Germany, Hungary, Luxembourg, Netherlands, Slovenia.

At Charité (the largest transplant center in Germany), living-donor programs were suspended briefly during the first wave of the pandemic. Although the deceased-donor program continued, ‘high-risk’ transplantations (i.e., extended-criteria donors, recipients with comorbidities, or donor–recipient partnerships exhibiting immunological incompatibilities) were not performed. In Italy, 2020 transplantation rates were predicted to match previous years, but with fewer procedures performed in the north (where the pandemic had greatest impact) and more in the south. In the US, living-donor programs paused (some for several months): kidney transplant volume was at 97.5% in 2020 compared with 2019, with an increase in deceased donors but fewer living donors (1, 2, 6). In the UK, all five London centers stopped kidney transplantations for months, although some regional centers continued operating (7). European participants reported no changes in recipient-selection protocols.

By November 2020, although all transplantations were resuming, there were backlogs of non-urgent cases, with many patients presenting late or with complex needs. To improve efficiencies, innovative care-delivery practices were being trialled, including new enhanced recovery pathways (reducing the hospital length of stay post-transplantation to ∼3 days, with remotely delivered aftercare (8). Discussants commented that such practices could become standard of care, if safety, financial efficiencies, and positive patient experiences were objectively demonstrated.

## Telemedicine

Early phases of the pandemic saw strong uptake (and acceptance) of telemedicine (9–11). Some discussants spoke of immediate efficiencies when consultations moved to virtual platforms, including reduced travel burden for staff and patients. Despite rapid implementation, telemedicine was well received, perhaps unexpectedly. However, virtual consultations could be technologically challenging, and therefore time consuming. Disparities in access to care were also evident: some patients did not own suitable devices or could not easily express themselves. Difficulties in maintaining patient confidentiality were mentioned, because clinicians could not influence patients’ locations for virtual appointments. Such consultations were therefore considered useful by the group, but were not expected to be adopted permanently. Finally, several discussants had difficulties obtaining reimbursement for telemedicine services.

Nevertheless, the value of telemedicine in the work-up and monitoring of kidney transplant recipients was noted. For example, at Charité, if recently discharged patients did not input daily blood pressure and temperature data they were automatically contacted; the clinical team could intervene, if necessary (9).

Although the pandemic created uncertainty for transplant patients, general advice about COVID-19 exposure, shielding, or procedural delays could be provided efficiently online [e.g., Massachusetts General Hospital (United States), videos: https://www.youtube.com/watch?v=hbrFLzbVFTA; https://www.youtube.com/watch?v=RP7clFVsYsk) (12)].

## Hospital Capacity

Even countries modestly affected by COVID-19 in 2020 had to consolidate health care. The pandemic overwhelmed intensive care unit (ICU)/high dependency unit (HDU) services in some regions such that other urgent-care capacity reduced by 50%. In the UK and elsewhere, centers that stopped transplantations redeployed clinical staff to other acute services. Discussants hoped that complete shutdowns would be avoided in future because reactivating departments (and associated research) was extremely challenging.

The pandemic disrupted the close collaboration that often develops between specialist transplant centers and individual patients. To comply with stay-in-place directives, many patients could only receive in-person care from local ‘general’ hospitals or community clinics. Although this had potential drawbacks in terms of continuity of care, discussants noted that it created new bonds between specialist and generalist centers and is a model to explore further.

## Transplant Candidate Selection

Discussants considered ethical aspects of performing kidney transplantations (particularly high-risk procedures) when ICU/HDU capacity is limited. While they agreed was appropriate to focus on low-risk transplantations when pressure on resources was greatest, the clinical dilemmas that this created should be acknowledged.

Of note, CMV-positive donor kidneys remained acceptable for CMV-negative recipients during the pandemic, but with greater emphasis on patient counselling (about transplantation risks and the importance of undergoing all scheduled post-procedural assessments).

## CMV Risk Management

Saving the graft remained the main aspect of CMV risk management. CMV prophylaxis, using agents with low side-effect profiles (avoiding leukopenia and neutropenia, in particular) was vital, to limit unplanned hospitalizations and associated risks.

Some centers did not adjust CMV prevention protocols in 2020; others switched from pre-emptive therapy to antiviral prophylaxis, and some increased the duration of prophylaxis. Indeed, comprehensive antimicrobial prophylaxis for the first 6–12 months post-transplantation and more frequent monitoring of CMV viral load (with increased use of local testing) were instrumental in mitigating overall risk of CMV infection/reactivation. No group members treated CMV without knowing the patient’s viral load.

No biomarker is commonly used to assess/adjust CMV prophylaxis, predict risk of neutropenia or leukopenia: decisions are based on clinical judgment. Tests for cellular-mediated immunity can predict CMV risk (especially R+ transplantatioins) but are rarely implemented. In addition, the absolute lymphocyte count can indicate risk for CMV infection at the end of treatment. Discussants suggested that an algorithm should be developed, to indicate specific prophylaxis regimens for specific patient types.

Although CMV reactivation was anticipated in kidney transplant recipients with concomitant COVID-19 because of immunomodulator use, only low-level reactivations were observed in 2020. Discussants were unaware of any research to investigate this further.

## Preventing Other Viral Infections

Annual influenza vaccination remains strongly recommended for transplant recipients. However, research is needed to establish the number of hospitalizations and pneumonia cases prevented by vaccination, and to characterize efficacy and safety profiles of both influenza and COVID-19 vaccinations post-transplantation. In November 2020, before COVID-19 vaccinations were licensed, discussants felt it would be unlikely there would be an absolute requirement for COVID-19 vaccination before kidney transplantation (note that this has evolved, and many programs mandate pre-transplant vaccination).

In the context of unvaccinated kidney transplant patients, the unmet need for effective antiviral treatments (for COVID-19 and influenza in particular) remained. Treatments (especially oral agents) given during the initial biologically driven phase of COVID-19 might help to reduce the risk of poor outcomes associated with the immune-driven phase of infection.

## COVID-19 Risk in Kidney Transplant Populations

In November 2020 discussants commented that fewer kidney transplant candidates or recipients had contracted COVID-19 compared with general-population rates, possibly because the transplant community was well practiced in infection control and social distancing. Timing, duration and nature of stay-at-home directives differed internationally, therefore no conclusions could be drawn on their effectiveness, particularly among kidney transplant recipients.

Regarding COVID-19 screening, PCR testing (nasopharyngeal swabs) was recommended. Although reperfusion technology has extended the time between kidney retrieval and successful transplantation (up to 40 h), rapid diagnostics needed to be a 24/7 service, given that transplantations often occur outside standard hours.

## Treating COVID-19 Infection in Kidney Transplant Recipients

In Spain in 2020, the incidence of COVID-19 was reportedly higher in people receiving kidney dialysis in hospital than in transplant recipients, possibly because they had limited ability to self isolate; this was demonstrated in UK research (13). The clinical course of COVID-19 infection was similar in transplant and dialysis patients, and worse than in the general population (14–18). Some discussants said that COVID-19 infections were rare within 6 months post-transplantation, probably because of antimicrobial prophylaxis.

Kidney transplant recipients were at a higher risk of death from COVID-19-related complications compared with the general population (19). However, data from an Italian center, collected during the early phase of the pandemic, indicated that all kidney transplant recipients with concomitant COVID-19 survived when immunosuppression was maintained (or switched from mycophenolate mofetil to high-dose steroids) (20). Centers that withdrew immunosuppression reported high rates of rejection and 30% mortality. Similar findings were described by discussants from Spain and the US. COVID-19 mortality rates were lower for patients who were several years post-kidney transplant if they were hospitalized at centers that were also specialist transplantation centers (21).

Opportunistic infections were not of concern in ICU patients with COVID-19, even if they were kidney transplant recipients: those who died were generally many years post-transplantation, elderly and had comorbidities, the group commented.

## Impact of COVID-19 on Health Policy

In November 2020, the picture was similar across Europe and the US: treatment delays for non-COVID-19-related conditions created a backlog that discussants felt would stretch resources for years (22, 23).

Patients were presenting with later stages of non-COVID-19 diseases than would be expected (24, 25): some were reluctant to attend hospital because they feared nosocomial COVID-19 (26). Across Europe, a substantial increase in use of anti-depressants and sleeping tablets (often self-medicated) was also noted.

For end-stage kidney disease, the impact of maintaining patients on dialysis (because of lack of transplantation services) is considerable. Maintenance dialysis costs ∼€50–90 000 per year; kidney transplantation in the first year costs ∼€30–86 000 while the annual ongoing management of a functioning graft reduces substantially, to ∼€5–20 000 (27, 28).

## Budget Impact

COVID-19-related healthcare costs in 2020 anticipated to reach billions of Euros in Europe) were funded separately; no changes to standard budgets were anticipated for 2021. However, given the backlog of non-COVID-19 cases, discussants wanted to identify and implement more efficient practices.

They commented that some initiatives (e.g., shorter in-patient stays) might be less valuable than initially anticipated: for example, even if patients are discharged early, fewer procedures were being performed because of reduced hospital capacity, social distancing, staff absences, etc. After the pandemic, analyzing big data might help to identify which improvements provided genuine benefits for specific services, including transplantation. Such analyses have been successful in HIV, hepatitis C management and oncology. Big data might also determine the number of excess deaths caused by the pandemic more accurately.

## Drug Development

Health Authority drug evaluations generally continued during 2020: potential treatments for COVID-19 were fast-tracked but processes continued with equal rigor, suggesting that efficiencies could be retained, especially for urgent medical needs. However, other new-drug evaluations were de-prioritized, and slower development pathways for non-COVID-related treatments may create access delays at a time when efficient methods to reduce backlogs are urgently needed.

## Health-Related Quality of Life for Kidney Recipients

Discussants agreed that a universal instrument to measure health-related quality of life in kidney transplant recipients would be valuable. Standard questionnaires may not capture what is important for patients, particularly in extraordinary times.

## Post Script: October 2021 Reflections From the Group

Table 2 compares discussants’ views in 2020 and late 2021. Discussants did not expect the pandemic to have long-lasting impact, with many countries experiencing restrictions through October 2021 despite widespread vaccination campaigns. In November 2020, no one anticipated that the pandemic had yet to peak. The largest wave hit Germany in early 2021, severely stretching its health system for the first time: capacity was halved in university hospitals and staff were redeployed to COVID-19 wards. Of note, deceased-donor transplantations generally continued, but living-donor procedures were greatly reduced (in part, because of patients’ concerns about nosocomial COVID-19) (2, 29).

**TABLE 2:** Key reflections from the 2020 webinars and 2021 discussions

Aspect of care	Views, November 2020	Views, October 2021
Transplant activity	• Decisions to continue/reduce transplantations, and redeploy clinical team differed by region	• No transplant centers closed, but living donor procedures paused when COVID-19 admissions were high
	• Transplantation capacity reduced (focus on DCD transplantations)	• Transplantations generally at near-normal level
	• Complete cessation of transplant services to be avoided (difficult to restart)	• Most centers had a backlog of cases and increase in patients with complex needs
Process adaptations	• Telemedicine and shared care (with local hospitals) were successful, but not expected to become permanent	• All forms of telemedicine remain widely accepted (new normal)
	• Information provision via social media was efficient	• Patients reluctant to attend hospital (fear of infection)
	• Technology enabled remote patient monitoring (and rapid hospital discharge)	• Technology poverty remains of concern
	• Technology poverty and poor skills created care disparities	• Reimbursement issues unresolved in some countries
	• Reimbursement issues for telemedicine apparent	
Candidate selection	• Focus on low-risk transplantations was necessary, but ethically and clinically challenging	• Autonomy needed for transplantation centers, given the life-years lost for waitlisted candidates
CMV risk management	• D+/R– transplantations continued but with greater emphasis on risk-management and pre-transplant counseling	• Virological control at risk because of reduced access to care and/or poor adherence
	• Some centers switched from pre-emptive therapy to antiviral prophylaxis (or from 6 to 12 months’ prophylaxis), with frequent viral load monitoring	• Severe CMV-related disease in D+/R– transplants increased morbidity/mortality with concomitant COVID-19
	• CMV reactivation: not a concern for kidney recipients with concomitant COVID-19	
Infection prevention in transplant recipients	• Influenza vaccination mandatory	• Transplant recipients reluctant to attend in-person appointments, even when essential
	• No COVID-19 vaccine licensed	• Most centers require transplant candidates to be fully vaccinated (including COVID-19, influenza); some extend this to immediate family
	• Mixed views on whether COVID-19 vaccination would be mandatory for transplant candidates	• Initial mRNA vaccinations less effective in kidney recipients than in general population: numerous additional boosters required
COVID-19 risk/outcomes for infected kidney transplant recipients	• Lower incidence of infection in recipients than in general public (pre-COVID-19 social distancing/infection control habits may have been beneficial)	• Recipients at higher risk of death from infection than general population. Risk factors: older age, comorbidities, many years post-transplantation
	• Outcomes worse in recipients than general public. Risk factors: older age, comorbidities	
Unmet needs	• Big data analysis: How many people contracted COVID-19 (excess mortality)?	• How to adjust studies for lost protocol visits?
	• Understand safety/efficacy of practice modifications, especially telemedicine, shortened hospital stay, and community–hospital collaboration	• Efficacy and safety of vaccinations (influenza and COVID-19) in kidney transplant populations
	• Develop health-related quality of life tool, specific for kidney transplant recipients	• How to reduce the immense, ongoing pressure on all members of the healthcare team
	• Algorithm to individualize CMV prophylaxis would be beneficial	

Factors affecting decisions to suspend, redeploy or continue transplantation services require full evaluation (2); data on this remain extremely limited (30). Some publications have emphasized the need to provide autonomy to transplantation centers, even if there are stay-at-home directives, given the impact on life years lost for waitlisted candidates (2, 29).

In October 2021, a population-based study investigated effects of the pandemic on transplantations across 22 countries (2). The study corroborated many points and predictions made at the November 2020 webinars, cautioning that other factors (including natural disasters) also affected service delivery (2). However, the study showed a 19% decline in kidney transplantations in 2020 compared with 2019, which was the highest reduction for any solid organ (2). Rates of deceased donor kidney transplantations fell by 12%, and living-donor transplantations by 40%. For those waitlisted for a kidney in 2020, the pandemic was associated with ∼37,664 patient life-years lost (2). Difficulties undertaking living-donor transplantations and paired kidney exchange during the pandemic, given the many societal restrictions, were inevitable. It is to be applauded that so many transplant centers managed to reduce their backlogs and return to near-normal service in 2021.

In terms of CMV management, patients receiving pre-emptive therapy risked losing virological control mechanisms because of reduced access to care (31, 32). Some discussants reported that severe disease in D+/R–transplantations negatively affected morbidity and mortality in patients with concomitant COVID-19; data corroborate this (33). In addition, clinical-trial participants lost protocolized visits, which implied they became protocol deviations. It remains unclear how this might affect data reporting (34, 35).

Discussants said that patients remained reluctant to attend in-person appointments, even when essential, although telemedicine remained well received (11, 36–39). Nevertheless, data on the impact of telemedicine in kidney transplant populations specifically are lacking, and the group spoke of continuing inequalities/access barriers (38, 39). Reimbursement for telemedicine remains unresolved in many countries, although for successful implementation see Duettman et al. (11).

Hospital–community and hospital–diagnostic partnerships continue, but initiatives have not been objectively evaluated. Nevertheless, discussants felt that COVID 19-related challenges were well managed by kidney transplantation communities. A tremendous amount of patient/family education was distributed. One participant commented, “Transplant patients looked to their clinical specialists for guidance. A lot of what we went through reinforced the strong relationship between transplant recipients and their clinicians.” The group remain concerned, however, about patients living with long-term illness and isolation, lacking access to appropriate clinical or respite care.

Discussants also described the unprecedented levels of mental and physical stress affecting healthcare professionals, given the unrelenting workloads. They were particularly concerned about the negative impact of the pandemic on morale among nurses, and on undergraduate medical education, especially the lack of access to patients. Such issues have been explored outside transplantation (40).

On reflection, if the pandemic continues, to reduce impact on health services some discussants would call for earlier stay-at-home directives, citing that a German lockdown in late 2020 may have helped hospital services in early 2021. Discussants agreed that reallocation of transplantation resources is inevitable folowing any rise in infections and hospitalizations, but stricter adherence to public-health measures could reduce infection rates, and therefore reduce the need to divert resources.

COVID-19 vaccination programs have so far been extremely successful, rapidly reducing the rates of serious infection and death in 201. Primary and booster vaccination remains the highest priority globally, as well as among transplant patients and their families. COVID-19 vaccination is generally (not universally) mandatory before organ transplantation and the requirement may even extend to close family members. The burden placed on transplantation teams to inform, counsel, vaccinate, determine immune responses and administer boosters to patients is considerable.

Research is required to ascertain short- and long-term levels of COVID-19 immunity among transplant recipients (41–46), and how best to treat those with severe infections. Administering a monoclonal antibody has been suggested (21). Without robust clinical evidence, prevention remains key: kidney transplant recipients’ antibody responses are monitored frequently (44) and repeated mRNA vaccine boosters are administered (44–47), given that immunity often appears to be short lived in these patients (48).

In 2021, COVID-19 infections were managed similarly in kidney transplant recipients and in the general population (49, 50), although transplant recipients had heightened risk of poor outcomes (51). Given the risks inherent in commenting on any single publication, the authors encourage regular review of guidance from the US National Institutes of Health (47) and the European Medicines Agency (52).

To conclude, discussants were relieved that many nearl-normal services resumed in 2021, despite ongoing challenges of COVID-19. They await a time when activities settle within a new-normal, where there are opportunities to meet, reflect and educate in person, so that more can be learned from this important time in our history.

## Human Cost of the Pandemic: Post-script Acknowledgment

Maintaining transplantation services with the additional infection-control measures required for COVID-19 has been extremely challenging for healthcare professionals. The pandemic lowered staff morale, and increased levels of fatigue. In addition, many healthcare professionals have been severely infected with COVID-19: some have lost colleagues or loved ones, or been debilitated by the infection and its aftermath. Most transplant clinicians have seen a substantial number of transplant recipients succumb to COVID-19 infection. Discussants agreed that this has been a very emotional and challenging time for all.

## Data Availability

The original contributions presented in the study are included in the article. Further inquiries can be directed to the corresponding author.

## Abbreviations

CMV, cytomegalovirus; HDU, high dependency unit; ICU, intensive care unit.
